# IL-27 regulates the number, function and cytotoxic program of antiviral CD4 T cells and promotes cytomegalovirus persistence

**DOI:** 10.1371/journal.pone.0201249

**Published:** 2018-07-25

**Authors:** Ellen J. Wehrens, Kurt A. Wong, Ankan Gupta, Ayesha Khan, Chris A. Benedict, Elina I. Zuniga

**Affiliations:** 1 Division of Biological Sciences, University of California San Diego, La Jolla, California, United States of America; 2 Division of Immune Regulation, La Jolla Institute for Allergy and Immunology, La Jolla, California, United States of America; University of Iowa, UNITED STATES

## Abstract

The role of IL-27 in antiviral immunity is still incompletely understood, especially in the context of chronic viruses that induce a unique environment in their infected host. Cytomegalovirus (CMV) establishes a persistent, tissue localized infection followed by lifelong latency. CMV infects the majority of people and although asymptomatic in healthy individuals, can cause serious disease or death in those with naïve or compromised immune systems. Therefore, there is an urgent need to develop a protective CMV vaccine for people at-risk and identifying key regulators of the protective immune response towards CMV will be crucial. Here we studied mouse CMV (MCMV) in IL-27 receptor deficient animals (*Il27ra*^*-/-*^) to assess the role of IL-27 in regulating CMV immunity. We found that IL-27 enhanced the number of antiviral CD4 T cells upon infection. However, in contrast to a well-established role for CD4 T cells in controlling persistent replication and a positive effect of IL-27 on their numbers, IL-27 promoted MCMV persistence in the salivary gland. This coincided with IL-27 mediated induction of IL-10 production in CD4 T cells. Moreover, IL-27 reduced expression of the transcription factor T-bet and restricted a cytotoxic phenotype in antiviral CD4 T cells. This is a highly intriguing result given the profound cytotoxic phenotype of CMV-specific CD4 T cells seen in humans and we established that dendritic cell derived IL-27 was responsible for this effect. Together, these data show that IL-27 regulates the number and effector functions of MCMV-specific CD4 T cells and could be targeted to enhance control of persistent/latent infection.

## Introduction

Interleukin 27 (IL-27) is a heterodimeric cytokine comprised of the subunits Epstein-Barr virus-induced gene 3 (EBI3) and IL-27p28. IL-27 signals through the common signal transducer gp130, which is shared with IL-6 and members of the IL-6 cytokine family, and Il-27Rα (also known as WSX-1 or TCCR) that confers ligand specificity [1]. Downstream signaling mainly occurs through STAT-1 and STAT-3, but IL-27 can also induce phosphorylation of STAT-4 and STAT-5 [2]. IL-27Rα is highly expressed on T cells and early studies have mainly focused on the biological role of IL-27 signaling in CD4 and CD8 T cells. However, expression of IL-27Rα is widespread throughout the immune system, including B cells and innate cells, and IL-27 has been shown to have pleiotropic roles in immune function.

Initially IL-27 was identified as a pro-inflammatory cytokine promoting the differentiation of T helper 1 (Th1) cells both *in vitro* [3] and *in vivo* [4, 5] through STAT-1 signaling. IL-27 achieves this effect by inducing T-bet expression and its downstream target IL-12Rβ2, thereby enhancing responsiveness to IL-12 and subsequent IFNγ production [2, 6]. However, more recently IL-27 has also been shown to act as a negative regulator of ongoing immune responses during infection and autoimmune inflammation [7] and under these circumstances IL-27 can limit pro-inflammatory cytokine production by CD4 T cells, including IFNγ [8–12]. This opposing effect of IL-27 on CD4 T cell IFNγ production can be explained by its role in induction, rather than maintenance of Th1 responses [5] and differential downstream signaling in naïve versus activated CD4 T cells [13]. Other immune regulatory effects of IL-27 include restriction of IL-2 production [14] and inhibition of Th17 differentiation [15]. Moreover, IL-27 can promote production of the inhibitory cytokine IL-10 [16–21] and functional specialization and maturation of FoxP3^+^ regulatory T cells (Tregs) [22, 23]. Although this immune regulatory role of IL-27 restricts pathogen control in models of parasitic or bacterial infection [8–11], the outcome of IL-27 mediated immune regulation seems more variable in the context of viral infection. Whereas IL-27 does not impact immune control of murine parainfluenza Sendai virus (SeV) infection [24], early IL-27 can impair control of influenza infection [25]. Similarly, the presence of IL-27 delays early control of mouse hepatitis virus (JHMV) infection, but does not affect the development of eventual persistence [26]. In contrast, we have previously shown that in mice infected with the continuous systemically replicating lymphocytic meningitis virus (LCMV) Clone 13, IL-27R signaling promotes the accumulation of anti-viral CD4 T cells late post infection and is absolutely required for viral control. Indeed, mice deficient in IL-27 receptor (*Il27ra*^*-/-*^) fail to clear replicating virus and develop life-long viremia [27]. Given this highly context dependent role of IL-27 in viral infection, we questioned its function in the immune response to persistent/latent viruses that, after a prolonged period of site-restricted viral replication, establish lifelong latency to subvert immune clearance. Cytomegalovirus (CMV) is a canonical example of these persistent/latent viruses and is highly common in humans, with seroprevalance being >90% in South American, African and Asian regions and around 50% in the US and Western Europe [28]. Although asymptomatic in healthy individuals, CMV infection can pose a life threatening risk in immune compromised patients and congenitally infected infants [29]. As a result, CMV is considered a major health concern and intensive effort is put towards the development of a protective vaccine [30]. However, vaccination attempts so far have been unsuccessful and in order to develop effective immunization strategies, more information on the requirements and regulation of the natural protective immune response against CMV is required. Due to millions of years of co-evolution with their respective hosts, CMVs do not show cross-species replication. However, mouse CMV (MCMV) shows a highly conserved course of infection as well as induction of innate and adaptive host immunity similar to human CMV, and has been extremely instructive in helping to delineate key components of protective CMV immunity.

CMV establishes prolonged viral replication at mucosal sites, including the salivary glands, which contributes to horizontal transmission of the virus [31]. Importantly, in mice CD4 T cells have been shown to be absolutely required for eventual control of MCMV replication in the salivary glands [32–34]. The likely importance of CD4 T cells in controlling human CMV infection is supported by observations of reduced presence of virus-specific effector CD4 T cells correlating with the development of CMV disease [35] and prolonged shedding of virus in the urine of young children [36]. However, the factors controlling the development of a strong effector CD4 T cell response against CMV remain to be determined.

Here, we investigated whether IL-27 regulates the numbers and function of antiviral CD4 T cells upon MCMV infection and what the consequences are for viral control. We found that IL-27 promotes the accumulation of antiviral CD4 T cells and induces IL-10 production in these cells. We also show that Dendritic Cell (DC) derived IL-27 restricts a cytotoxic program in MCMV specific CD4 T cells, including the cytotoxic effector molecule granzyme A (GrzA). These IL-27 induced changes in effector CD4 T cell number and function are accompanied by impaired control of persistent MCMV replication in the salivary glands, indicating that IL-27 is an attractive target to modulate CD4 T cell responses and enhance control of CMV persistence.

## Results

### IL-27R signaling cell-intrinsically regulates the magnitude of the antiviral CD4 T cell response upon MCMV infection

**Fig 1:**
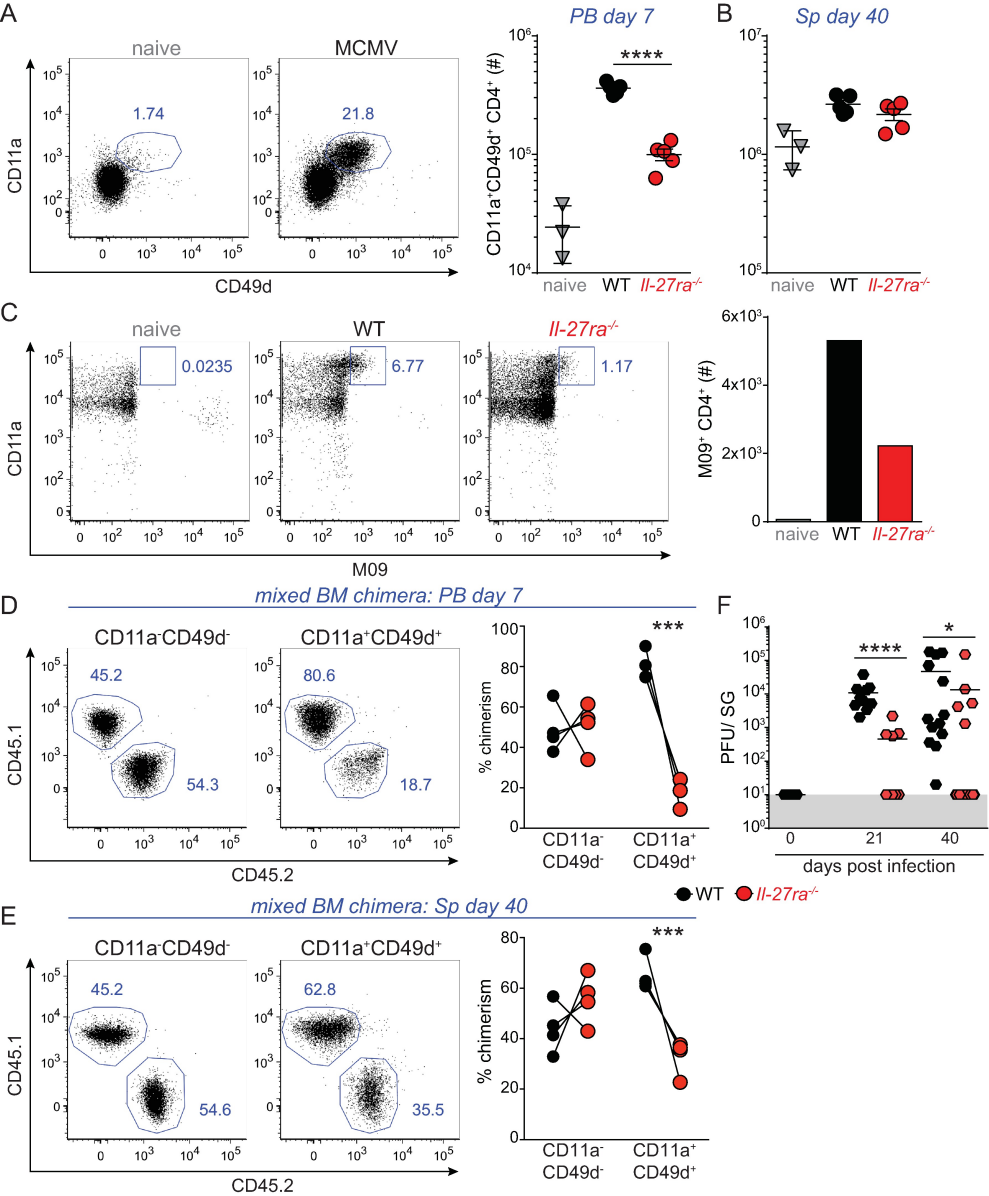
IL-27 receptor signaling enhances the number of antiviral CD4 T cells despite promoting MCMV persistence. WT and *Il27ra*^*-/-*^ mice were infected with 1*10^4^ PFU MCMV and MCMV-specific CD4 T cell responses were monitored. Representative dotplot showing the gating strategy based on combined expression of CD11a and CD49d and CD11a^+^CD49d^+^ CD4 T cells enumerated in the blood at day 7 p.i. (A) and spleen at day 40 p.i. (B). M09_133-147_ specific CD4 T cells were quantified using peptide-MHCII tetramer enrichment in pooled spleens at day 40 p.i. (C). (D-E) WT:*Il27ra*^*-/-*^ chimeric mice were infected with 1*10^4^ PFU MCMV and the proportions of CD11a^-^CD49d^-^ versus CD11a^+^CD49d^+^ CD4 T cells residing within the WT and *Il27ra*^*-/-*^ compartment were analyzed in the blood at day 7 p.i. (D) and in the spleen at day 40 p.i.(E). (F) Viral titers were determined in the salivary glands (SG) of WT and *Il27ra*^*-/-*^ mice at day 21 and day 40 p.i. (A-B) one representative of 3 independent experiments with n = 4–5 mice per group. (C) one representative of 2 independent experiments with pooled samples of n = 4–5 mice per group. (D-E) one representative of 2 independent experiments with n = 4–5 mice per group (F) Pooled data of 2–3 independent experiments with n = 4–5 mice per group. * p < 0.05, *** p < 0.001, **** p < 0.0001.

To investigate whether IL-27 regulates antiviral CD4 T cells during infection with the persistent/latent virus MCMV, we first investigated whether numbers of MCMV-specific CD4 T cells are altered in *Il27ra*^*-/-*^ mice, lacking the IL-27 specific component of the IL-27 receptor complex, compared to wildtype (WT) controls. With the exception of M09_133-147_ specific CD4 T cells that show delayed expansion at later time points post infection (p.i.) [37], expansion of CD4 T cells against all other previously identified MCMV epitopes peaks around day 7 p.i. [37] and can be identified polyclonally through co-expression of CD11a and CD49d [38]. When analyzing the amount of CD11a^+^CD49d^+^ CD4 T cells in the blood at day 7 p.i. we observed a significant drop in numbers in *Il27ra*^*-/-*^ mice compared to WT controls (Fig 1A). We also quantified antiviral CD4 T cells during persistent infection, when MCMV replication is restricted to the salivary glands after being cleared from systemic organs [32]. At this time point (day 40 p.i.) we did not see a difference in overall CD4 T cell responses identified by CD11a and CD49d in the spleen of *Il-27ra*^*-/-*^ mice compared to WT controls (Fig 1B). However, we did observe reduced numbers of M09_133-147_ specific CD4 T cells, known to dramatically increase towards day 40 p.i. [37], in pooled samples of *Il27ra*^*-/-*^ mice compared to WT controls (Fig 1C).

To investigate whether IL-27 intrinsically regulated the number of antiviral CD4 T cells upon infection, we generated WT:*Il27ra*^*-/-*^ chimeric mice by reconstituting sublethally irradiated recipients with a 1 to 1 ratio of WT and *Il27ra*^*-/-*^ bone marrow cells and tracked WT versus *Il-27ra*^*-/-*^ CD4 T cells at different times post infection based on congenic marker expression (S1A–S1C Fig). IL-27 mediated regulation of antiviral CD4 T cells became more apparent under competitive conditions, with almost the entire polyclonal CD11a^+^CD49d^+^ antiviral CD4 T cell population residing within the WT compartment of chimeric mice. This was highly evident at the time of peak expansion of these cells in the blood at day 7 p.i. (Fig 1D), but also apparent in the spleen at day 40 p.i. (Fig 1E). In contrast, the proportion of CD11a^-^CD49d^-^ CD4 T cells (Fig 1D and 1E, left panels), as well as the total CD4 T cell population before and after infection (S1A–S1C Fig), was equally distributed between the WT and *Il-27ra*^*-/-*^ compartment. This demonstrates that there is no overall reconstitution advantage of WT over *Il-27ra*^*-/-*^ CD4 T cells in uninfected mice, and that IL-27R signaling specifically and cell-intrinsically regulates the MCMV-specific CD4 T cell response. Due to this strong IL-27 mediated regulation of antiviral CD4 T cells under competitive conditions, levels of *Il-27ra*^*-/-*^ CD11a^+^CD49d^+^ CD4 T cells upon MCMV infection in chimeric mice did not exceed the numbers observed in uninfected controls (S1D Fig). As a result, it is unclear whether the relatively small numbers of *Il-27ra*^*-/-*^ CD11a^+^CD49d^+^ CD4 T cells in chimeric mice represent true MCMV-specific CD4 T cells or merely background levels of activated cells also observed in uninfected mice, which compromised further phenotypical and functional comparison of WT and *Il-27ra*^*-/-*^ CD11a^+^CD49d^+^ CD4 T cells in chimeric mice.

Interestingly, IL-27 appeared to play a less prominent role in regulating the magnitude of the CD8 T cell response, with no consistent differences in antigen experienced cells co-expressing CD11a and CD49d seen in the blood at day 7 p.i. (S2A Fig) nor in the spleen at day 40 p.i. (S2B Fig), even under competitive conditions in chimeric mice (S2C Fig). We also analyzed M45_985-993_ and M38_316-323_ specific CD8 T cells that are prototypic of CD8 T cells that contract and persist at low frequencies after resolution of acute infection versus inflationary populations that increase after acute infection before stabilizing at high frequency, respectively [39]. Both M45_985-993_ and M38_316-323_ positive CD8 T cells reside within the CD11a^+^CD49d^+^ population analyzed in S2B Fig and numbers of these epitope specific CD8 T cells were also not different in spleens from WT versus *Il27ra*^*-/-*^ mice at day 40 p.i. (S2D and S2E Fig). Thus, while unessential for regulating antiviral CD8 T cell numbers, intrinsic IL-27R signaling positively regulates the number of MCMV specific CD4 T cells upon infection and specifically ‘late-rising’ M09_133-147_ reactive CD4 T cells that are immunodominant during the persistent phase of MCMV infection.

### IL-27 promotes persistence of MCMV in the salivary glands

Inability of antigen presenting cells (APCs) to cross-present viral antigens and lack of MHC-I expression on infected cells due to immune evasion [34] allows MCMV to escape CD8 T cell recognition in the salivary gland. As such, CD4 T cells are absolutely required to curtail, and eventually resolve, persistent viral replication in this organ [32, 34]. Moreover, M09_133-147_ specific CD4 T cells, which expand during the late-persistent phase of MCMV infection [37], play a key role in regulating the duration of MCMV replication in salivary glands (A. Gupta, G. Picarda and C. Benedict, submitted). Given that IL-27 promoted the numbers of antiviral CD4 T cells upon infection, and specifically M09_133-147_ specific CD4 T cells, we reasoned that IL-27 signaling would also help control viral replication in the salivary glands. To investigate this, we quantified viral titers in the salivary glands of WT and *Il27ra*^*-/-*^ mice at day 21 and day 40 p.i. Unlike what we have previously shown for systemic persistent infection with LCMV Cl13 [27], viral titers were dramatically reduced in *Il27ra*^*-/-*^ mice, with the majority of animals already clearing the virus at times when persistent replication was still ongoing in WT hosts (Fig 1F), in line with a recent study by Clements et al. [40]. Overall, our data indicate that despite a critical, cell-intrinsic role of IL-27Rα in promoting accumulation of MCMV specific CD4 T cells, IL27Rα signaling actually restricted control of MCMV persistence in salivary glands.

### IL-27 induces IL-10 production in CD4 T cells during MCMV infection

**Fig 2:**
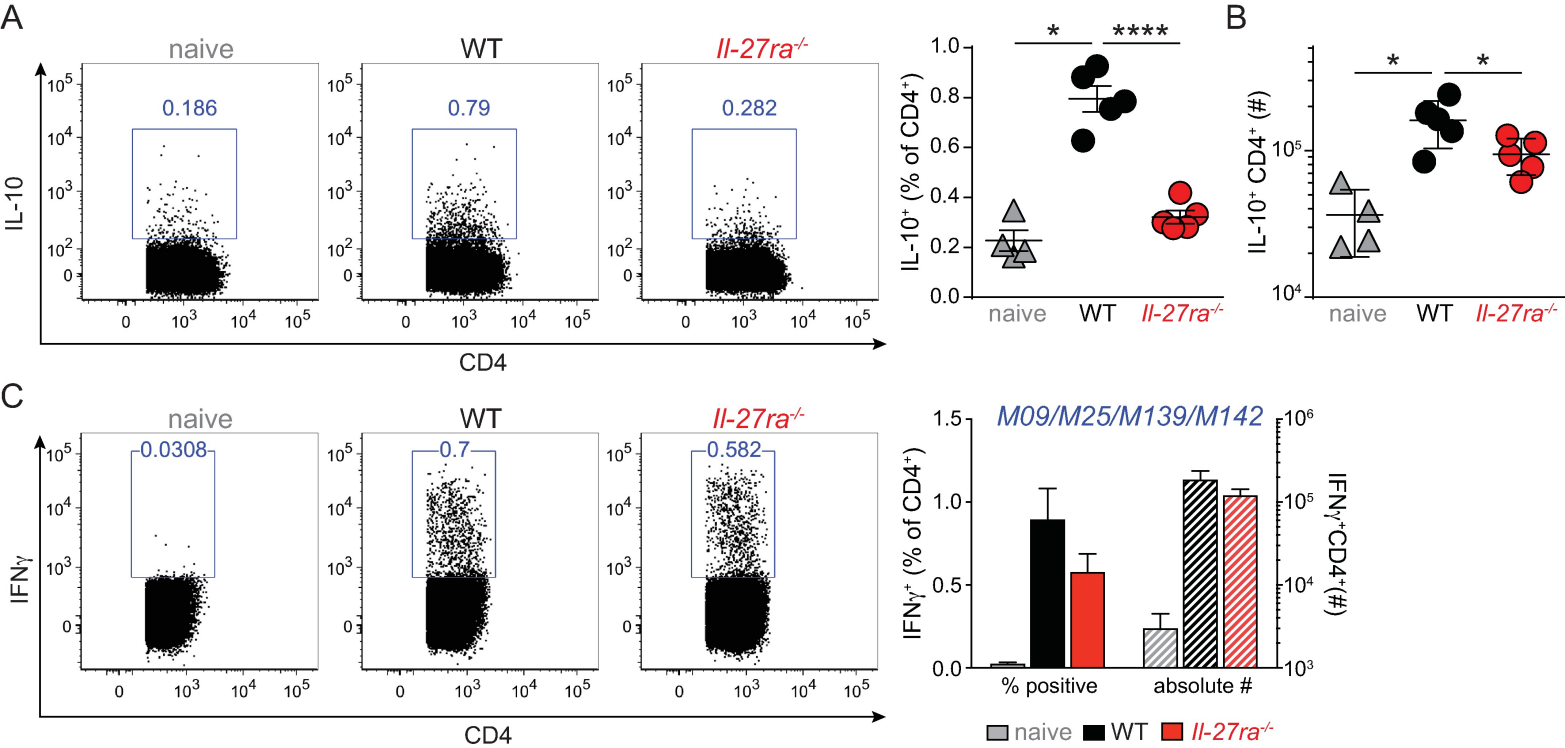
IL-27 induces IL-10 production by CD4 T cells upon MCMV infection. WT and *Il27ra*^*-/-*^ mice were infected with 1*10^4^ PFU MCMV. The proportion (A) and number (B) of IL-10 producing CD4 T cells upon PMA/ion stimulation were analyzed in the spleen at day 21 p.i. (C) The proportion and number of IFNγ producing CD4 T cells were determined upon M09_133-147_, M25_409-423_, M139_560-574_ and M142_24-38_ peptide specific restimulation in the spleen at day 40 p.i. (A-B) one representative of two independent experiments with n = 5 mice per group. (C) one representative of 3 independent experiments with n = 4–5 mice per group * p < 0.05, **** p < 0.0001.

Control of persistent replication in the salivary glands has been shown to be dependent on IFNγ production by CD4 T cells [34, 41], but restricted by IL-10 [42]. Therefore changes in either IFNγ or IL-10 levels could underlie the improved viral control noted in the absence of IL-27 signaling. To investigate this, we first compared the presence of IL-10 producing CD4 T cells in WT and *Il27ra*^*-/-*^ mice infected with MCMV. In line with previous work establishing IL-27 as a potent inducer of IL-10 in CD4 T cells [17, 21] and recent results from *Il27ra*^-/-^ mice infected with MCMV [40], we found a significant reduction in the proportion (Fig 2A) and number (Fig 2B) of CD4 T cells that produced IL-10 in *Il27ra*^*-/-*^ mice. During *in vitro* or *in vivo* Th1 polarizing conditions, IL-27 mediated induction of IL-10 in CD4 T cells is mostly restricted to IFNγ producing cells [17, 19] also considered T regulatory 1 cells [16]. Indeed, the proportion (S3A Fig) and number (S3B Fig) of IL-10 producing cells was similarly reduced in *Il27ra*^*-/-*^ mice when analyzed within IFNy producing CD4 T cells.

In mice lacking IL-10 expression in all cells (*Il10*^*-/-*^) [43], as well as in T cell specific IL-10 deficient mice (*CD4*^*cre*^ *Il10*^*fl/fl*^) [40] and upon *in vivo* IL-10R blockade [42], absence of IL-10 signaling enhances the number of IFNγ producing CD4 T cells upon MCMV infection. Also in *Il27ra*^*-/-*^ mice, it was recently noted that reduced IL-10 production by CD4 T cells coincided with enhanced CD4 IFNγ responses [40]. However, when analyzed in the spleen at day 40 p.i., we did not observe an increase in the proportion or number of CD4 T cells producing IFNγ upon specific re-stimulation with a pool of CD4 restricted MCMV peptides (Fig 2C). In addition, the proportion (reaching statistical significance in two out of three independent experiments), but not numbers, of IFNγ^+^ CD4 T cells were reduced upon polyclonal PMA/ion stimulation (S3C Fig). Since we observed an impact of IL-27 on the number of antiviral CD4 T cells present upon infection (Fig 1), we also normalized numbers of IFNγ producing CD4 T cells to the number of polyclonal CD11a^+^CD49d^+^ antiviral CD4 T cells detected. Again, we observed no difference in this normalized number of IFNγ^+^ CD4 T cells between WT and *Il27ra*^*-/-*^ mice (S3D Fig). Moreover, the amount of IFNγ produced per cell responding to either pooled MCMV peptide (S3E Fig) or PMA/ion stimulation (S3F Fig) (analyzed by means of MFI in IFNγ positive cells) did not differ between WT and *Il27ra*^*-/-*^ mice. Thus, we did not find evidence for enhanced CD4 T cell IFNγ production in *Il27ra*^*-/-*^ mice, even when accounting for the reduced number of antiviral CD4 T cells present in these mice. This finding is contrary to recent work by Clements et al. [40] and might be related to the time post infection analyzed and/or specific restimulation method used to induce IFNγ production. However the absence of a clear reduction in CD4 IFNγ responses in the presence of a striking increase in viral control (Fig 1F) also raises the interesting possibility that other functional changes in CD4 T cells might contribute to the enhanced viral control *in Il-27ra*^*-/-*^ mice.

### IL-27R signaling restricts a cytotoxic phenotype in MCMV specific CD4 T cells

**Fig 3:**
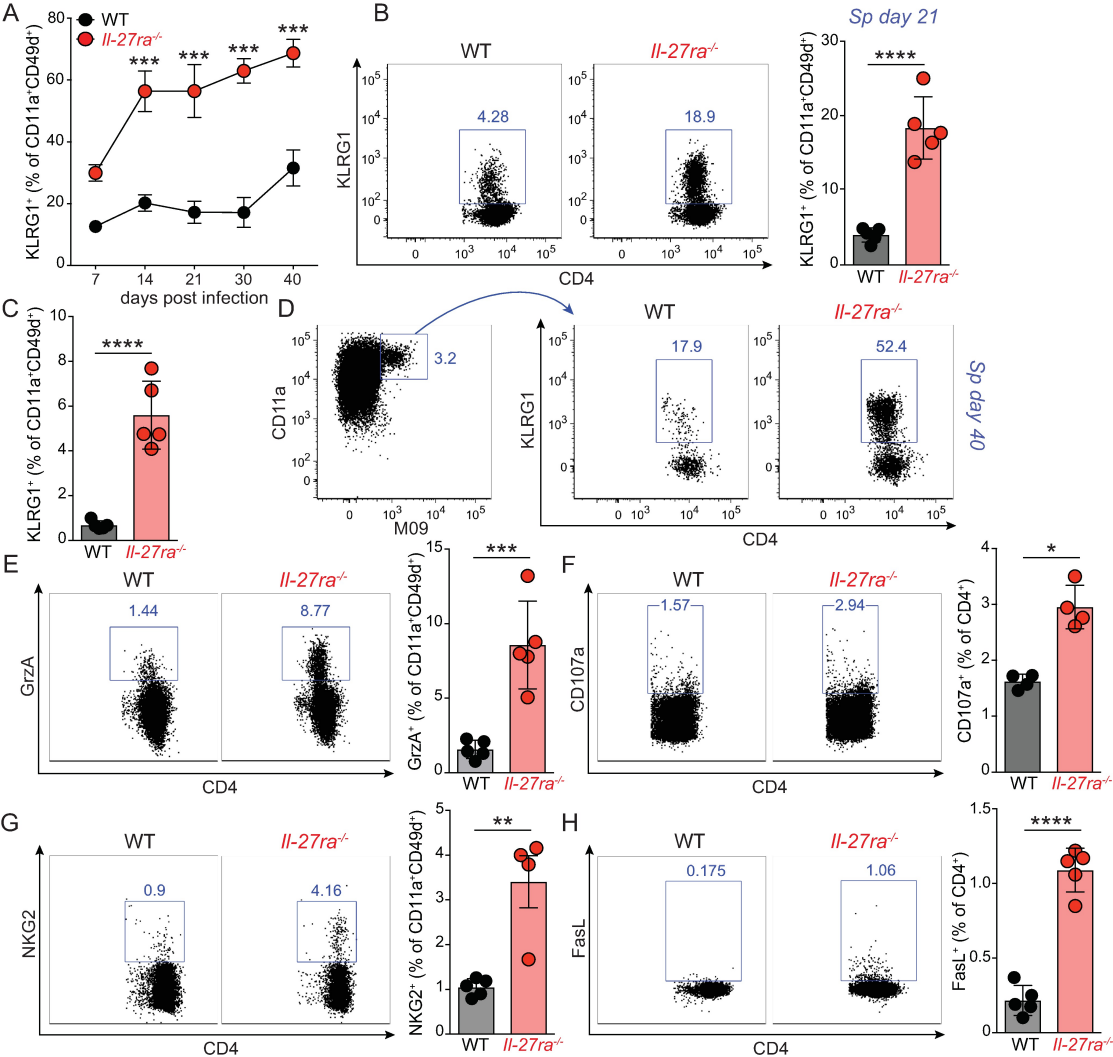
IL-27 restricts the development of MCMV-specific CD4 T cells displaying a cytotoxic phenotype. WT and *Il27ra*^*-/-*^ mice were infected with 1*10^4^ PFU MCMV and the proportion of polyclonal CD11a^+^CD49d^+^ CD4 T cells expressing KLRG1 were analyzed throughout the course of infection in the blood (A) and in the spleen at day 21 (B) and day 40 p.i. (C). (D) The proportion of KLRG1 expressing M09_133-147_ specific CD4 T cells in the spleen at day 40 p.i. (E) Proportion of CD11a^+^CD49d^+^ CD4 T cells expressing granzyme A (GrzA) in the spleen at day 21 p.i. (F) The proportion of CD4 T cells that express CD107a upon PMA/ion stimulation in the spleen at day 7 p.i. (G-H) Proportion of polyclonal CD11a^+^CD49d^+^ CD4 T cells expressing NKG2D (G) and FasL (H) in the spleen at day 21 p.i. All data are representative of at least 2 independent experiments with n = 4–5 mice per group. * p < 0.05, ** p < 0.01, *** p < 0.001, **** p < 0.0001.

To further characterize changes in antiviral CD4 T cells in the absence of IL-27R signaling during MCMV infection, we phenotyped polyclonal CD11a^+^CD49d^+^ CD4 T cells at multiple time points post infection in the blood and observed a dramatic increase in the proportion of cells that expressed KLRG1 in *Il27ra*^*-/-*^ mice as early as day 14 p.i., which was maintained throughout the course of infection (Fig 3A). This increase in KLRG1 expressing cells was also observed in polyclonal CD4 T cells analyzed in the spleen at day 21 p.i. (Fig 3B) and in both polyclonal (Fig 3C) and inflationary M09_133-147_ specific CD4 T cells (Fig 3D) at day 40 p.i.

Given that KLRG1 expression in CD4 T cells has been associated with acquisition of cytotoxic function [44], we next compared antiviral CD4 T cells from WT and *Il27ra*^*-/-*^ mice for the expression of cytotoxicity-associated markers. Killing of target cells can be achieved through the release of cytolytic granules containing granzymes. Expression of granzyme B was low in splenic MCMV specific CD4 T cells, as we saw previously [38], and did not differ between *Il27ra*^*-/-*^ and WT animals (data not shown). However, the proportion of granzyme A expressing CD11a^+^CD49d^+^ CD4 T cells was markedly enhanced in *Il27ra*^*-/-*^ mice (Fig 3E). In addition, at day 7 p.i. (but not at later time points) a small, but significantly higher proportion of CD4 T cells from *Il27ra*^*-/-*^ mice expressed CD107a on the cell surface, suggesting that an increased fraction of the cells underwent cellular degranulation [45] (Fig 3F). We also observed increased expression of NKG2 receptors (A/C/E) on antiviral CD4 T cells from *Il27ra*^*-/-*^ mice (Fig 3G), which specifically mark cytotoxic CD4 T cells in murine influenza A infection [46]. A second effector mechanism employed by cytotoxic CD4 T cells depends on binding of Fas Ligand (FasL) to its cognate receptor Fas on the target cells, inducing caspase-3-mediated apoptosis [47]. This cytotoxic function might also be enhanced in antiviral CD4 T cells from *Il27ra*^*-/-*^ mice, as suggested by a small, but consistent increased proportion of FasL expressing cells (Fig 3H). Of note, this increased proportion of cells with a cytotoxic phenotype did not result from a reduced presence of other non-cytotoxic subsets, but reflected an accumulation of this population, indicated by enhanced absolute numbers of CD11a^+^CD49d^+^ CD4 T cells expressing KLRG1 (S4A Fig), GrzA (S4B Fig), NKG2A/C/E (S4C Fig) and FasL (S4D Fig) in *Il-27ra*^*-/-*^ mice.

### The cytotoxic phenotype of MCMV-specific CD4 T cells correlates with IL-27 regulated T-bet expression

**Fig 4:**
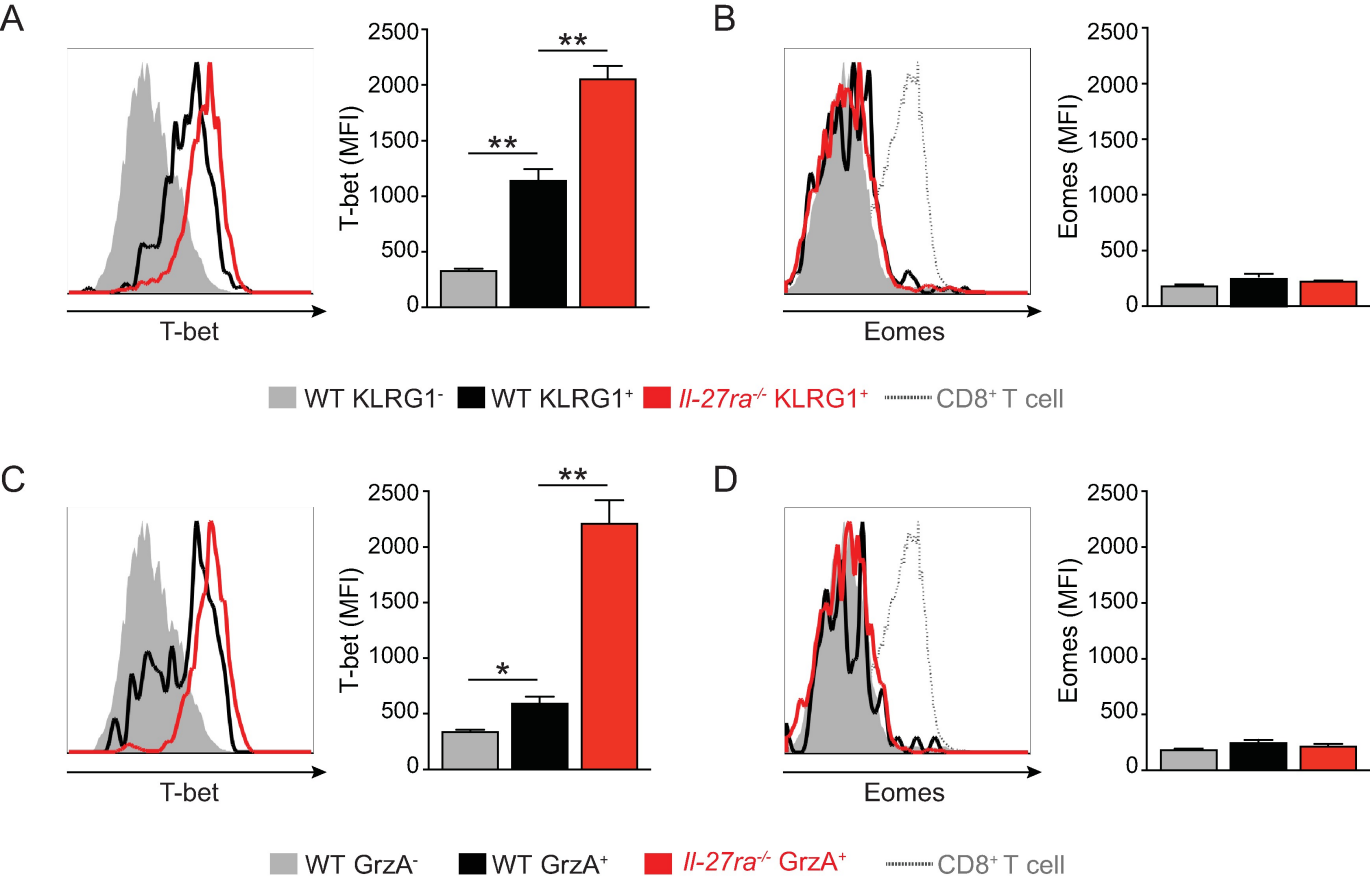
IL-27 inhibits T-bet expression in antiviral CD4 T cells with a cytotoxic phenotype. WT and *Il27ra*^*-/-*^ mice were infected with 1*10^4^ PFU MCMV and expression of the transcription factors T-bet and Eomes was analyzed in CD11a^+^CD49d^+^ CD4 T cells by mean fluorescence intensity (MFI) at day 21 p.i. (A-B) T bet (A) and Eomes (B) expression in KLRG1^-^ and KLRG1^+^ cells from WT animals compared to KLRG1^+^ cells from *Il27ra*^*-/-*^ mice. (C-D) T-bet (C) and Eomes (D) expression in GrzA^-^ and GrzA^+^ cells from WT animals compared to GrzA^+^ cells from *Il27ra*^*-/-*^ mice. As a positive control for Eomes staining, CD11a^+^CD49d^+^ CD8 T cells were included in histograms depicting Eomes expression (dotted line). One representative of 2 independent experiments with n = 5 mice per group. * p < 0.05, ** p < 0.01.

Cytotoxic CD4 T cells have classically been viewed as functional variants of Th1 cells [48] and indeed the Th1 associated transcription factor T-bet can drive expression of cytotoxic genes [49, 50]. However, more recently cytotoxic CD4 T cells have been suggested to form a separate CD4 T helper subset, depending on linage specific transcription factors [48, 51, 52], including Eomes [44, 53, 54], which is thought to be essential for both their differentiation and function. We observed that during MCMV infection CD4 cytotoxic phenotype highly correlated with T-bet, but not Eomes expression. Accordingly, T-bet mean fluorescence intensity (MFI) was significantly increased in KLRG1^+^ compared to KLRG1^-^ CD11a^+^CD49d^+^ CD4 T cells from WT MCMV infected mice and was further increased in KLRG1^+^ cells from *Il27ra*^*-/-*^ animals (Fig 4A). In contrast, Eomes expression was low in both KLRG1^-^ and KLRG1^+^ CD11a^+^CD49d^+^ CD4 T cells, even when analyzed in KLRG1^+^ cells from *Il-27ra*^*-/-*^ mice (Fig 4B). A similar correlation was observed for T-bet and GrzA expression: T-bet MFI was increased in GrzA^+^ versus GrzA^-^ CD11a^+^CD49d^+^ CD4 T cells from WT mice and further upregulated in GrzA^+^ cells in the absence of IL-27Rα signaling (Fig 4C). Again Eomes expression was equally low in all three cell populations analyzed (Fig 4D), but not in CD11a^+^CD49d^+^ CD8 T cells analyzed in parallel as a positive control for Eomes staining (dotted line in histograms Fig 4B and 4D). Together, these data show that during MCMV infection, the development of a cytotoxic phenotype in CD4 T cells correlates with expression of the transcription factor T-bet and not Eomes, and accordingly IL-27 negatively regulates T-bet expression in CD4 T cells upon infection.

### Dendritic cell derived IL-27 is required to restrict a cytotoxic phenotype in CD4 T cells during MCMV infection

To our knowledge, IL-27 mediated restriction of a cytotoxic program in antiviral CD4 T cells has not been described before and we sought to identify the relevant cellular source for this new role of IL-27 during infection. Myeloid cells, including macrophages, inflammatory monocytes and DCs are considered dominant sources of IL-27 production [7]. Therefore, we used mice with an IL-27p28 conditional allele (*Il27p28*^*fl/fl*^) crossed to *CD11c*^*cre*^ mice, to specifically delete IL-27p28 in DCs [55]. In addition, we used the same *IL27p28*^*fl/fl*^ strain crossed to *LysM*^*cre*^ mice to delete IL-27p28 in macrophages, neutrophils and monocytes [56]. Both *CD11c*^*cre*^ and *LysM*^*cre*^ *Il27p28*^*fl/fl*^ mice were infected with MCMV and we first confirmed cell type specific deletion of *Il27p28* by FACS purifying innate immune populations at 36 hrs p.i. (S5A and S5B Fig) and determining the relative levels of *Il-27p28* by qPCR. In line with published literature [56], *LysM*^*cre+*^ *Il27p28*^*fl/fl*^ mice showed partial deletion of *Il-27p28* (43–47%) in monocytes (S5C Fig) and a profound reduction (76–89%) in neutrophils (S5D Fig). In *CD11c*^*cre+*^ *Il27p28*^*fl/fl*^ mice we also observed partial deletion of *Il-27p28* in monocytes (S5E Fig), but most importantly, and in contrast to *LysM*^*cre+*^ *Il27p28*^*fl/fl*^ mice (S5F Fig), an almost complete deletion of *Il-27p28* in both CD11b^+^ (77–85%) and CD8^+^ (96–97%) conventional DCs (cDCs) (S5G Fig). Of note, both genetic models showed the same overall deletion of *Il-27p28* systemically, as measured in spleen homogenates at 36 hours p.i. (S5H and S5I Fig). Having confirmed the cell type specific deletion of *Il-27p28* in both genetic models, we next, analyzed KLRG1 and GrzA expression at different time points post infection to investigate which cellular source was important for IL-27 mediated regulation of a cytotoxic phenotype in antiviral CD4 T cells.

**Fig 5:**
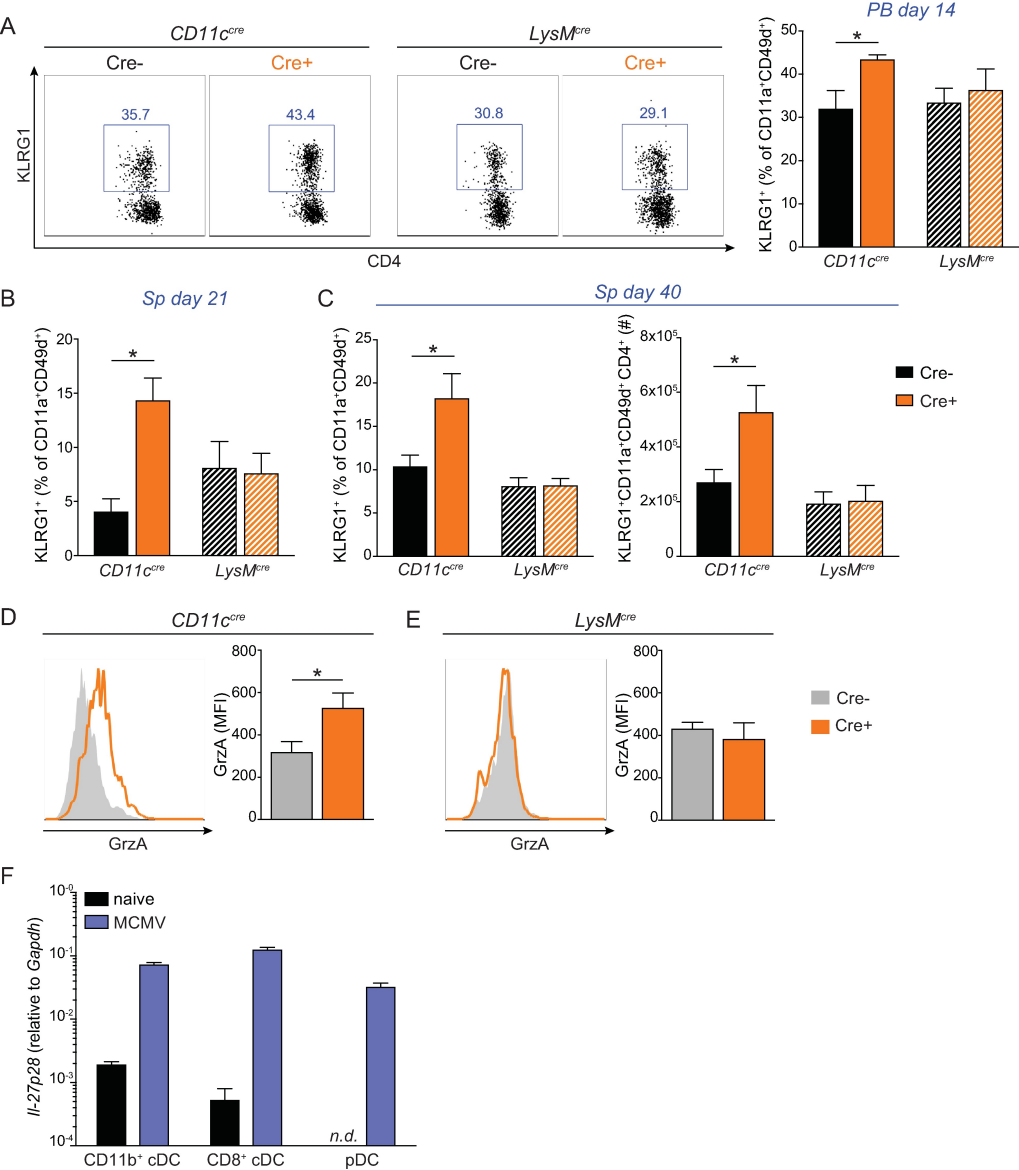
IL-27 produced by DCs upon infection restricts a cytotoxic phenotype in antiviral CD4 T cells. *CD11c*^*cre+*^*IL27p28*^*fl/fl*^, *LysM*^*cre+*^*IL27p28*^*fl/fl*^ and cre- littermate controls were infected with 1*10^4^ PFU MCMV and KLRG1 expressing CD11a^+^CD49d^+^ CD4 T cells were analyzed in the blood at day 14 p.i. (A) and in the spleen at day 21 (B) and day 40 (C) p.i. (D-E) GrzA mean fluorescence intensity (MFI) in CD11a^+^CD49d^+^ CD4 T cells from *CD11c*^*cre+*^*IL27p28*^*fl/fl*^ mice (D) and *LysM*^*cre+*^*IL27p28*^*fl/fl*^ (E) compared to cre- littermate controls in the blood at day 14 p.i. (F) *Il27p28* transcript levels relative to *Gapdh* in FACS purified CD11b^+^ cDCs (PI^-^Thy1.2^-^CD19^-^NK1.1^-^CD138^-^CD11c^hi^B220^-^CD11b^+^CD8^-^), CD8^+^ cDCs (PI^-^Thy1.2^-^CD19^-^NK1.1^-^CD138^-^CD11c^hi^B220^-^CD11b^-^CD8^+^), and pDCs (PI^-^Thy1.2^-^CD19^-^NK1.1^-^CD138^-^CD11c^dim^CD11b^-^B220^+^PDCA^+^) from spleens of WT mice 36 hours post MCMV infection compared to uninfected controls. (A-E) representative of two independent experiments with n = 5 mice per group. (F) One independent experiments with pooled samples from n = 5 mice per group. * p < 0.05.

When CD11a^+^CD49d^+^ antiviral CD4 T cells were analyzed in the blood at day 14 p.i., we observed an increased proportion of KLRG1 expressing cells in *CD11c*^*cre+*^ *IL27p28*^*fl/fl*^ mice compared to *Cre-* littermate controls, but not in *LysM*^*cre+*^ animals (Fig 5A). The same result was obtained at later time points post infection in the spleen with increased CD11a^+^CD49d^+^ CD4 T cells expressing KLRG1 in *CD11c*^*cre*+^, but not *LysM*^*cre+*^ mice compared to *Cre*- controls at day 21 (Fig 5B) and day 40 p.i (Fig 5C). Again KLRG1 expression correlated with cytotoxic molecule expression, as shown by increased expression of GrzA by CD11a^+^CD49d^+^ CD4 T cells in the blood of *CD11c*^*cre+*^ mice compared to littermate controls (Fig 5D), but not *LysM*^*cre+*^ mice compared to *Cre*- littermates (Fig 5E). Given that both genetic models showed the same deletion of *Il-27p28* in monocytes and systemically in the spleen (S5C, S5E, S5H and S5I Fig), but only *CD11c*^*cre+*^ mice exhibited profound deletion of *Il-27p28* in DCs (S5F and S5G Fig), these results indicated that it was the specific lack of *Il-27p28* in DCs (S5G Fig) that dictated the enhanced CD4 cytotoxic phenotype in *CD11c*^*cre+*^ *Il27p28*^*fl/fl*^ mice.

Given the biological relevant role of DC-derived IL-27 in limiting a cytotoxic phenotype in antiviral CD4 T cells, we next further evaluated the subsets of DCs producing IL-27 after MCMV infection. In line with S5F and S5G Fig, we again found that in uninfected mice CD11b^+^ and CD8^+^ cDCs expressed basal levels of *Il27p28* transcript and this was highly increased upon MCMV infection (Fig 5F). Notably, although in our experimental conditions we could not detect *Il27p28* transcript in plasmacytoid DCs (pDCs) from uninfected mice, they highly upregulated *Il27p28* upon MCMV infection (Fig 5F). Together, these data show that IL-27 is produced by multiple DC subsets (i.e. pDCs, CD11b^+^ and CD8^+^ cDCs) after MCMV infection and that DC-derived IL-27 is required to restrict a cytotoxic phenotype in antiviral CD4 T cells upon infection. In contrast, although IL-27 was found to be produced by monocytes and neutrophils upon MCMV infection (S5C and S5D Fig), IL-27 derived from these cells appears to be dispensable for its role in restricting a cytotoxic phenotype in antiviral CD4 T cells.

## Discussion

IL-27 is a pleiotropic cytokine with multiple roles in immune function. Although it can promote the initial activation of naïve T cells, it can also down-regulate their activity and effector functions during ongoing immune responses *in vivo*, including bacterial [10] and parasitic infection [8, 9, 11, 12]. This role of IL-27 in restraining T cell activation can enhance parasitic [8, 11] or bacterial [10] burden, but is also necessary to prevent T cell mediated immune pathology [8, 11] and in some cases even mortality [8–10, 12]. IL-27’s role in the immune response to viral infection seems to be more context dependent with varying outcomes on viral control and immune pathology depending on the class of virus [24–27].

Here, we investigated the role of IL-27 upon infection with MCMV, a persistent/latent virus, and similar to our previous results with the persistent systemically replicating virus; LCMV Cl13 [27], found that IL-27 positively regulated the numbers of antiviral CD4 T cells, as noted by reduced numbers of polyclonal CD11a^+^CD49d^+^ and M09_133-147_ specific CD4 T cells in *Il27ra*^*-/-*^ mice. The reduction in polyclonal CD4 T cells was already evident at the peak of acute infection (day 7 p.i.), in line with a role for IL-27 in promoting early expansion of CD4 T cells upon activation [3, 57]. IL-27 might mediate this effect by inhibiting cell death, similar to what has been shown in a colitis model [58] and as we reported for CD4 T cells deficient in the IL-27 co-receptor gp130 during chronic LCMV infection [27]. Notably, WT:*Il27ra*^*-/-*^ chimeric mice showed a dramatic reduction in antiviral CD4 T cells in the *Il27ra*^*-/-*^ compartment, but not in overall CD4 T cell reconstitution, demonstrating that Il-27R signaling cell-intrinsically regulates antiviral CD4 T cell numbers and that WT virus-specific CD4 T cells have a substantial competitive advantage over *Il27ra*^*-/-*^ cells within the same infectious environment. Interestingly, evidence for IL-27 playing the same prominent role in regulating antiviral CD8 T cell numbers was not observed, and this might result from lower IL-27Ra expression by CD8 T cells compared to CD4 T cells, as has been reported under steady state conditions [4, 5, 59]. Despite positively regulating CD4 T cell numbers, including M09_133-147_ specific CD4 T cells, which are particularly important in regulating MCMV persistence (A. Gupta, G. Picarda and C. Benedict, submitted), IL-27Rα signaling promoted persistent replication of MCMV in the salivary glands, in line with a recent publication by Clements et al. [40]

Control of viral replication in the salivary gland has been shown to be dependent on CD4 IFNγ production, but restricted by IL-10 [42], and indeed a reduced proportion of CD4 T cells from *Il27ra*^*-/-*^ mice produced IL-10. However, in our experiments this reduction in IL-10 producing CD4 T cells was not accompanied by an increase in IFNγ producing CD4 T cells. This is contrary to what has been shown for both genetic knock down of IL-10 [40, 43] and IL-10R blocking antibody treatment [42] in MCMV infected mice, and in a recent paper studying MCMV infection in *Il27ra*^*-/-*^ mice [40]. This discrepancy could stem from differences in experimental set-up; including the time post infection analyzed, dose and/or viral isolate used for MCMV infection and/or the use of pooled epitopes for re-stimulation rather than individual peptides. Although it remains to be determined that CD4 T cells are responsible for the enhanced clearance observed in *Il-27ra*^*-/-*^ mice, the requirement for these cells in the eventual clearance of persistent replication in WT animals [32–34] makes this a highly likely possibility. Our findings suggest that additional functional changes in antiviral CD4 T cells, besides IFNγ production, may play a role in the enhanced control of MCMV persistence in *Il-27ra*^*-/-*^mice. In favor of this hypothesis, it has been shown that recombinant IFNγ cannot substitute for CD4 T cells in clearing persistent replication from the salivary glands [41] and MCMV utilizes the M27 viral protein to restrict IFNγR signaling [60]. In addition, although the absence of IL-27 consistently reduces IL-10 producing CD4 T cells in multiple types of viral infections, the outcome for viral control is highly variable [24–26]. Thus IL-27’s impact on controlling viral infection seems to extend beyond its downstream mediator IL-10.

By further characterizing antiviral CD4 T cells in the absence of IL-27R signaling, we demonstrated a dramatic increase in the proportion of cells expressing KLRG1 that was maintained throughout the course of infection. These data are in line with increased KLRG1 expression by CD4 T cells from *Il27ra*^*-/-*^mice infected with malaria, although in this model KLRG1 expression correlated with more terminally differentiated Th1 cells, producing higher amounts of IFNγ [61], which we did not observe. Instead, we noted increased expression of multiple cytotoxic markers in antiviral CD4 T cells from *Il27ra*^*-/-*^ mice, in line with a previous report identifying KLRG1 as a marker for cytotoxicity [44]. This is highly intriguing since in human CMV infection, antiviral CD4 T cells develop a very strong cytotoxic phenotype [62–64] and are capable of killing antigen-loaded target cells *ex vivo* [64]. Moreover, the direct effector function(s) of CD4 T cells, as opposed to B cell and T cell help [35], and specifically their cytolytic capacity [65], correlates with reduced CMV disease in renal transplant recipients. In contrast to CD8 T cells, for which it is considered prototypic, cytotoxicity has much more recently been recognized as a relevant function of CD4 T cells with far reaching implications, not only for infectious disease, but also autoimmune disease and cancer [48, 66, 67]. However, a lot still remains to be discovered about the *in vivo* requirement allowing for cytotoxic CD4 T cell differentiation. Our data add to this exciting new field by demonstrating that *in vivo* IL-27Rα signaling restricts the expression of cytotoxic molecules in CD4 T cells during the persistent phase of MCMV infection, the most important phase for horizontal transmission of this virus.

Although initially considered functional variants of Th1 cells, the identification of lineage specific transcription factors [48, 51, 52], including Eomes [44, 53, 54], has now led to the assumption that cytotoxic CD4 T cells form a separate subset of CD4 T cells [48]. However, our data show that during MCMV infection the expression of cytotoxic molecules by antiviral CD4 T cells correlated with T-bet and not Eomes expression. Accordingly, we found that IL-27 restricted T-bet and not Eomes levels in KLRG1^+^ or GrzA^+^ CD4 T cells. This down-regulation of T-bet is contrary to IL-27 promoting T-bet expression in naive CD4 T cells [3], but in line with what has been shown during malaria infection [12] and probably results from its preferential induction of STAT3 (rather than STAT1) in activated cells [13, 27]. In addition, these data suggest that the development of cytotoxic CD4 T cells during MCMV infection might be a feature of Th1 cells regulated through T-bet and not Eomes. In line with this it has been shown that T-bet can bind to the promoter of cytotoxic genes and promote their transcription [49, 50]. Moreover, our preliminary data showed similar expression of cytotoxic molecules in MCMV specific CD4 T cells from CD4-ER-Cre Eomes^fl/fl^ mice in which Eomes was deleted specifically in CD4 T cells prior to infection (unpublished observation). In contrast, Eomes does directly regulate CD4 cytotoxic molecule expression upon LCMV Cl13 infection [68].

A severe reduction in CD11a^+^CD49d^+^ CD4 T cells developing from the *Il27ra*^*-/-*^ compartment of chimeric mice complicated the analysis of a direct role for IL-27Rα signaling in regulating CD4 T cell cytotoxic phenotype, and it remains to be determined whether IL-27 regulates CD4 cytotoxic phenotype through intrinsic or extrinsic mechanisms. Published data shows that a cell-extrinsic increase in KLRG1 is unlikely to be secondary to reduced IL-10 production in *Il27ra*^*-/-*^ mice, as *Il10*^*-/-*^ and *Il10r*^*-/-*^ mice show no (or a very marginal) upregulation of KLRG1 upon infection [12]. Instead, IL-27 [22] and specifically DC derived IL-27 [69] has been demonstrated to induce a specific population of CXCR3^+^T-bet^+^ Treg, specialized to limit Th1 cells [70] and we found significantly reduced proportions of these cells in *Il27ra*^*-/-*^ mice, although numbers were not different, due to enhanced overall numbers of FoxP3^+^ Tregs (S6 Fig). These data suggest that a relative reduction of CXCR3^+^T-bet^+^ Th1 like Tregs might contribute to the enhanced cytotoxic phenotype of effector Th1 cells in our model. Thus, the IL-27 mediated restriction of a cytotoxic program in antiviral CD4 T cells could result from combined direct and indirect regulatory mechanisms. IL-27 could directly suppress effector Th1 cells, most likely through STAT3 signaling, as well as promoting the functional maturation of FoxP3^+^ Treg that are now better equipped to suppress Th1 effector cells. We also showed that down-regulation of CD4 cytotoxic molecule expression required DC derived IL-27, whereas IL-27 production by monocytes and neutrophils appeared to be dispensable. All DC subsets; CD11b^+^ cDCs, CD8^+^ cDCs and pDCs quickly upregulated *Il-27p28* transcript upon MCMV infection, similar to what we recently reported upon LCMV Cl13 infection [71].

Although CMV is asymptomatic in healthy individuals, it can pose a severe threat in naïve and immune compromised patients, including congenitally infected infants and solid organ transplant recipients. As such the development of an effective CMV vaccine has been listed a top health priority [30]. Natural immunity to CMV can protect against disease-causing secondary infections and it is therefore important to understand the factors controlling natural immunity to CMV and incorporate them into vaccine design. Our evidence that IL-27 promotes CMV persistence and, in addition to promoting IL-10 production, down-regulates T-bet expression and restricts a cytotoxic program in antiviral CD4 T cells, strongly suggest that this cytokine should be considered in the context of CMV vaccine development.

## Materials and methods

### Mice and viral stocks

WT C57BL/6J, B6.SJL-*Ptprc*^*a*^*Pepc*^*b*^/BoyJ (CD45.1) and *Il27ra*^*-/-*^ mice were purchased from the Jackson Laboratory. *CD11c*^*cre*^*Il27p28*^*fl/fl*^ and *LysM*^*cre*^*Il27p28*^*fl/fl*^ mice were generously provided by Dr. L. Lu (UCSD). Mice were housed and bred under specific-pathogen-free conditions with maximum 5 mice per cage, food and water provided ad libitum and nesting material added for shelter. Mice were infected i.p. with 1x 10^4^ PFU of salivary gland derived Smith Strain of MCMV. Titration of MCMV stock and replication levels in salivary glands were determined by plaque assay in 3T3 cells as previously described [38]. MCMV infection did not cause significant health complications or distress as measured by changes in weight loss, activity level and physical appearance. Mice were euthanized using CO2 inhalation followed by cervical dislocation as a secondary physical method to ensure death. Mixed bone marrow chimeras were generated by transferring a 1:1 ratio of bone marrow cells from WT CD45.1 and *Il27ra*^*-/-*^ CD45.2 mice into lethally irradiated CD45.1 mice as previously described [72]. To avoid secondary infections upon irradiation, mice were treated two weeks with Sulfamethoxazole and Trimethoprim supplemented in the drinking water.

### Ethics statement

All experiments were approved by the Institutional Animal Care and Use Committee (IACUC) at UCSD under protocol S07315. Animal procurement, housing, and handling conformed to the National Institutes of Health (NIH) Guide for the Care and Use of Laboratory Animals in Research (DHEW 80–23), all requirements of the United States Department of Agriculture (USDA) and all regulations issued by the USDA implementing the Animal Welfare Act (P.L. 89–544).

### Cell purification

Spleens were incubated with 1mg/ml collagenase D (Roche) for 20 min at 37°C and passed through a 100μm strainer to achieve a single cell suspension. Red blood cell lysis of both spleen and blood samples was performed using ACK buffer. For qPCR analysis, splenocytes were enriched for DCs with PanDC microbeads using an Automacs system (Miltenyi) or depleted of T and B cells using Thy1.2 (clone 53–2.1) and CD19 (6D5) biotinylated antibodies and EasySep Mouse Streptavidin RapidSpheres (Stemcell technologies). Enriched fractions were then stained with flow cytometry antibodies alongside propidium iodide (PI) and FACS purified using a BD ARIA II (BD Biosciences).

### Peptide-MHC-II tetramer enrichment

Biotinylated Class II M09_133-147_ (I-A^b^) monomer was provided by the NIH Tetramer Facility and tetramerized by addition of Streptavidin-APC (Life Technologies) according to their protocol. Single cell suspensions from pooled spleens were subjected to M09_133-147_ tetramer enrichment with slight modifications of a previously described protocol [73]. In brief, splenocytes were incubated with 10 μg/ml of tetramer for 90 min at room temperature, followed by incubation with anti APC beads (Miltenyi) for 20 min at 4°C. Subsequently, samples were enriched for bead-bound cells on a magnetic column (LS, Miltenyi) and stained for flow cytometry.

### Flow cytometry

The following antibodies purchased from BD Biosciences, eBioscience or BioLegend were used to stain blood or spleen cells: B220 FITC, A780 or PE-CF594 (all clone RA3-6B2) were used to exclude B cells from analysis. CD11a PE or BV510 (clone M17/4), CD49d Pcp-E710 or Biotin (clone R1-2) followed by Streptavidin PE-CF594, CD4 BV650 or A700 (clone RM4-5) and CD8 A700, PB or A780 (all clone 53–6.7) were used to stain antigen experienced CD4 and CD8 T cells. For analysis of CD4 T cells, Tregs were excluded using FoxP3 APC or FITC (FJK-16s). Virus specific CD8 T cells were stained using biotinylated Class I M38_316-323_ (K-b) and M45_985-993_ (D-b) monomer provided by the NIH Tetramer Facility, which were tetramerized using Streptavidin-APC (Life Technologies) and incubated at 10 μg/ml for 20 min at 4˚C. CD45.1 BV605 (clone A20) and CD45.2 BV650 (clone 104) were used to distinguish between the WT (CD45.1) and *Il-27ra*^*-/-*^ (CD45.2) compartment of chimeric mice. Cytokine analysis was performed using IL-10 PE (clone JESS-16E3) and IFNγ APC (XMG1.2). The following markers were used to analyze CD4 T cell cytotoxic phenotype: KLRG1 FITC or PE-Cy7 (both clone 2F1), GrzA PE-Cy7 or APC (clone GzA-3G8.5), CD107a PcP-E710 (eBio1D4B), NKG2A/C/E PcP-E710 (20d5), FasL (CD178) PE (MFL3) and the transcription factors T-bet PE (4B10) and Eomes PE-eFluor610 (Dan11mag). To phenotype Treg, FoxP3 E450 (FJK-16s), T-bet PE (4B10) and CXCR3 APC (CXCR3-173) were used. The following antibodies were used for FACS sorting of innate immune populations: Thy1.2 PcP-E710 (30-H12) or PE (53–2.1), CD19 PcP-Cy5.5 or PE (both eBio1D3), NK1.1 PcP-Cy5.5 or PE (both PK136), GR-1 PcP-Cy5.5 (RB6-8C5), Siglec-F PcP-E710 (1RNM44N), Streptavidin PcP-Cy5.5 and CD138-PE (281–2) to exclude non-DC and non-monocyte lineages. CD11c APC (N418), CD11b PE-Cy7 (M1/70), CD8 FITC or PcP-Cy5.5 (both clone 53–6.7), B220 A780 (RA3-6B2), PDCA FITC (eBio927), GR-1 FITC (RB6-8C5) and Ly6C A780 (HK.1.4) to positively select monocytes, neutrophils and DC subsets. Purified Rat Anti-Mouse CD16/32 (2.462) was used to block non-specific binding to Fc receptor expressing cells and Propidium iodide solution (Sigma-Aldrich) to exclude non-viable cells. Internuclear permeabilization and staining for transcription factors was performed using the FoxP3 staining kit (eBioscience) according to the manufacturer’s instructions. For detection of intracellular cytokines, cells were fixed using 1% paraformaldehyde and permeabilized using saponin (Sigma). Viable cells were gated using Live/Dead Aqua (Life Technologies). Samples were acquired on a BD LSRII (BD Biosciences) and analyzed using FlowJo software (TreeStar).

### Ex vivo T cell stimulation

For peptide specific re-stimulation, splenocytes were cultured in RPMI 1640 supplemented with 2mM L-glutamine, 100 U/ml penicillin-streptomycin and 10% FBS for a total of 8 hours in the presence of 3μg/ml each of a pool of MHC class-II restricted peptides (M09_133-147_, M25_409-423_, M139_560-574_ and M142_24-38_ (all >95% pure; A&A labs) with addition of Brefeldin A (1.25 μg/ml; Sigma) for the last 6 hours of culture. For polyclonal stimulation, splenocytes were cultured for 5 hours with PMA (10 ng/ml; Sigma), Ionomycin (0.5 μg/ml; EMD Millipore) and Brefeldin A (1.25 μg/ml; Sigma). To evaluate cell degranulation CD107a PcP-E710 (eBio1D4B) was added to PMA/ion stimulated cultures.

### Real time quantitative PCR

Total RNA from sorted cells was extracted using RNeasy Micro kits (Qiagen) and reverse transcribed into cDNA using Superscript III RT (Invitrogen). Tissue disruption of spleen homogenates was performed using Qiashredder columns (Qiagen), RNA extracted with RNeasy Mini kits (Qiagen) and reverse transcribed into cDNA using M-MLV (Promega). *Il27p28* quantification was performed using SYBR green PCR kits (Applied Biosystems) on a Real-Time PCR Detection System (Applied Biosystems or BioRad) and normalized to *Gapdh* using the following primers; *Il27p28*: F 5’-CTGTTGCTGCTACCCTTGCTT-3’ R 5’-CACTCCTGGCAATCGAGATTC-3’ (C57BL/6 mice) or *Il27p28 (expand exon3/4)* F 5’-CTGAATCTCGATTGCCAGGAGTGA-3’ R 5’-AGCGAGGAAGCAGAGTCTCTCAGAG-3’ (*CD11c*^*cre*^ and Lysm^*cre*^*Il27p28*^*fl/fl*^ mice) and *Gapdh*: F 5’- TCCCACTCTTCCACCTTCGA-3’ R 5’-AGTTGGGATAGGGCCTCTCTT-3’.

### Statistical analysis

Comparison of two groups was performed using Student t test. Paired t test was used to compare the *Il27ra*^*-/-*^ compartment to the WT compartment of chimeric mice. In case of non-Gaussian distribution, data were tested non-parametrically, using Mann Whitney-U test or Wilcoxon matched-pairs signed rank test, respectively. For comparison of 3 or more groups ANOVA or non-parametric Kruskal-Wallis test were used followed by Sidak’s multiple comparisons test or non-parametric Dunn’s multiple comparisons test to compare selected pairs of groups. All analyses were performed using GraphPad Prism 6 software. * p < 0.05, ** p < 0.01, *** p < 0.001, **** p < 0.0001.

## Supporting information

S1 Fig Specific lack of CD11a^+^CD49d^+^ CD4 T cells in the *Il27ra*^*-/-*^ compartment of infected chimeras. WT:*Il27ra*^*-/-*^ chimeric mice were left untreated or infected with 1*10^4^ PFU MCMV and the proportion of CD4 T cells residing within the WT (CD45.1^+^) versus *Il27ra*^*-/-*^ (CD45.2^+^) compartment was determined in the blood prior to infection (A), in the blood at day 7 p.i. (B) and in the spleen at day 40 p.i. (C). (D) The proportion of CD11a^+^CD49d^+^ CD4 T cells in the WT and *Il27ra*^*-/-*^ compartment of MCMV infected compared to uninfected chimeric mice. One representative of 2 independent experiments with n = 4–5 mice per group. *** p < 0.001, *ns* = not significant.
(TIF)

S2 Fig IL-27 receptor signaling does not regulate the number of virus-specific CD8 T cells. WT and *Il27ra*^*-/-*^ mice were infected with 1*10^4^ PFU MCMV and antigen experienced CD11a^+^CD49d^+^ CD8 T cells were enumerated in the blood at day 7 p.i. (A) and spleen at day 40 p.i. (B). (C) WT:*Il27ra*^*-/-*^ chimeric mice were infected with 1*10^4^ PFU MCMV and the proportion of CD11a^+^CD49d^+^ CD8 T cells within each compartment was analyzed in the blood (PB) of both uninfected and MCMV infected chimeric mice (day 7 p.i.) and in the spleen (Sp) of infected chimeras at day 40 p.i. (D-E). WT and *Il27ra*^*-/-*^ mice were infected with 1*10^4^ PFU MCMV and the number of M45_985-993_ (D) and M38_316-323_ (E) virus specific CD8 T cells was determined in the spleen at day 40 p.i. (A-B) one representative of 3 independent experiments with n = 4–5 mice per group. (C-E) one representative of 2 independent experiments with n = 4–5 mice per group. *ns* = not significant.
(TIF)

S3 Fig IL-27 induces IL-10 in IFNγ^+^CD4 T cells, but does not restrict IFNγ production. WT and *Il27ra*^*-/-*^ mice were infected with 1*10^4^ PFU MCMV and the proportion (A) and number (B) of IL-10^+^ cells within IFNγ producing CD4 T cells was analyzed upon PMA/ion stimulation in the spleen at day 21 p.i. (C) WT and *Il27ra*^*-/-*^ mice were infected with 1*10^4^ PFU MCMV and the proportion and number of IFNγ producing CD4 T cells were determined upon PMA/ion stimulation in the spleen at day 40 p.i. (D) The number of IFNγ producing CD4 T cells upon M09_133-147_, M25_409-423_, M139_560-574_ and M142_24-38_ peptide specific restimulation and polyclonal PMA/ion stimulation in the spleen at day 40 p.i. normalized for the total amount of polyclonal CD11a^+^CD49d^+^ CD4 T cells present. (E-F) IFNγ mean fluorescence intensity (MFI) in IFNγ^+^ CD4 T cells upon M09_133-147_, M25_409-423_, M139_560-574_ and M142_24-38_ peptide specific restimulation (E) and polyclonal PMA/ion stimulation (F) in the spleen at day 40 p.i. All data are representative of at least two independent experiments with n = 4–5 mice per group. * p < 0.05, ** p < 0.01, *** p < 0.001.
(TIF)

S4 Fig IL-27 restricts the number of CD4 T cells that display a cytotoxic phenotype upon infection. WT and *Il27ra*^*-/-*^ mice were infected with 1*10^4^ PFU MCMV and the number of polyclonal CD11a^+^CD49d^+^ CD4 T cells expressing KLRG1 (A), GrzA (B), NKG2A/C/E (C) and FasL (D) were determined in the spleen. All data are representative of at least two independent experiments with n = 5 mice per group. ** p < 0.01, ****p < 0.001.
(TIF)

S5 Fig Cell type specific deletion of *Il27p28* in *LysM*^*cre+*^*IL27p28*^*fl/fl*^ and *CD11c*^*cre+*^*IL27p28*^*fl/fl*^ mice. *LysM*^*cre+*^*IL27p28*^*fl/fl*^ and *CD11c*^*cre+*^*IL27p28*^*fl/fl*^ mice or cre- littermate controls were left untreated or infected with 1*10^4^ PFU MCMV. 36 hours post infection innate cell populations were FACS purified from pooled spleen samples and the relative levels of *Il27p28* over *Gapdh* were determined by qPCR. (A-B) gating strategy and post sort purity in *LysM*^*cre+*^*IL27p28*^*fl/fl*^ mice (A) and *CD11c*^*cre+*^*IL27p28*^*fl/fl*^ mice (B). Relative level of *Il27p28* over *Gapdh* in sorted monocytes (PI^-^Thy1.2^-^CD19^-^NK1.1^-^Siglec-F^-^CD11c^-^CD11b^hi^Ly6C^hi^) (C), neutrophils (PI^-^Thy1.2^-^CD19^-^NK1.1^-^Siglec-F^-^CD11c^-^CD11b^hi^GR-1^+^) (D) and cDCs (PI^-^Thy1.2^-^CD19^-^NK1.1^-^Siglec-F^-^CD11c^hi^) (F) of infected *LysM*^*cre+*^*IL27p28*^*fl/fl*^ mice compared to cre- and uninfected controls. Relative level of *Il27p28* over *Gapdh* in sorted monocytes (PI^-^Thy1.2^-^CD19^-^NK1.1^-^Siglec-F^-^GR-1^-^CD11c^-^CD11b^hi^) (E) and CD11b^+^ (PI^-^Thy1.2^-^CD19^-^NK1.1^-^Siglec-F^-^GR-1^-^CD11c^hi^CD11b^+^CD8^-^) and CD8^+^ (PI^-^Thy1.2^-^CD19^-^NK1.1^-^Siglec-F^-^GR-1^-^CD11c^hi^CD11b^-^CD8^+^) cDCs (G) of infected *CD11c*^*cre+*^*IL27p28*^*fl/fl*^ mice compared to cre- and uninfected controls. (H-I) *Il27p28* transcript levels relative to *Gapdh* in spleen homogenates of *LysM*^*cre+*^*IL27p28*^*fl/fl*^ mice (H) and *CD11c*^*cre+*^*IL27p28*^*fl/fl*^ mice (I) at 36 hours p.i. compared to cre- and uninfected controls One representative of two independent experiments with pooled samples from n = 4–5 mice per group.
(TIF)

S6 Fig IL-27 promotes CXCR3^+^T-bet^+^ FoxP3^+^ Tregs upon MCMV infection. WT and *Il27ra*^*-/-*^ mice were infected with 1*10^4^ PFU MCMV. (A) Gating strategy applied to quantify CD4^+^CXCR3^+^T-bet^+^FoxP3^+^ Tregs. Proportion (B) and number (C) of CD4^+^CXCR3^+^T-bet^+^FoxP3^+^ Tregs and total number of CD4^+^FoxP3^+^ Tregs (D) analyzed in the spleen at d21 p.i. All data are representative of two independent experiments with n = 5 mice per group. * p < 0.05, ** p < 0.01.
(TIF)
