# Supplementary figures and images for: IL-27 regulates the number, function and cytotoxic program of antiviral CD4 T cells and promotes cytomegalovirus persistence
Source: PLoS One. 2018 Jul 25;13(7):e0201249. doi: 10.1371/journal.pone.0201249 (PMC6059457; doi:10.1371/journal.pone.0201249)

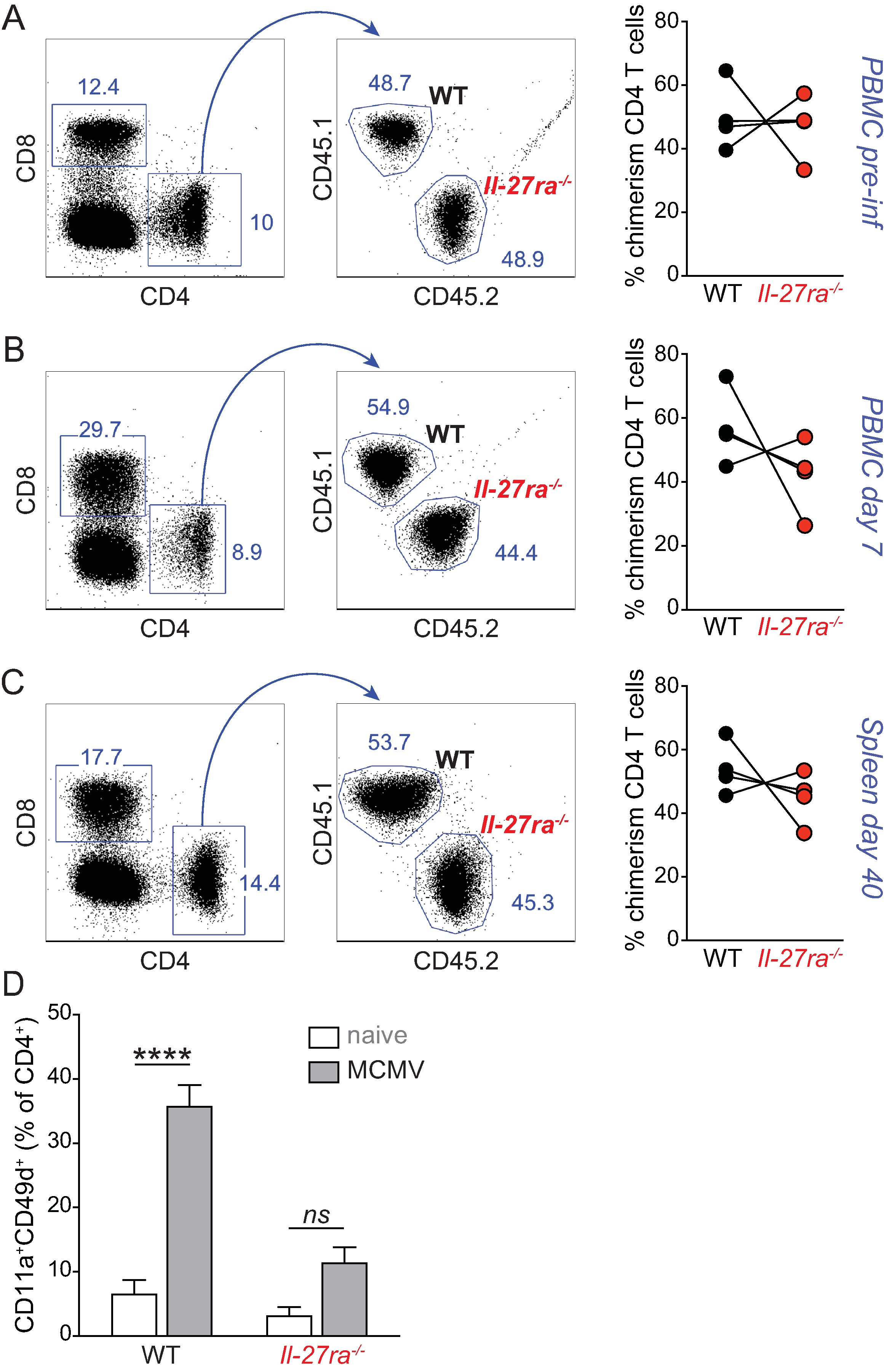

Supplement: S1 Fig — WT:Il27ra-/- chimeric mice were left untreated or infected with 1*104 PFU MCMV and the proportion of CD4 T cells residing within the WT (CD45.1+) versus Il27ra-/- (CD45.2+) compartment was determined in the blood prior to infection (A), in the blood at day 7 p.i. (B) and in the spleen at day 40 p.i. (C). (D) The proportion of CD11a+CD49d+ CD4 T cells in the WT and Il27ra-/- compartment of MCMV infected compared to uninfected chimeric mice. One representative of 2 independent experiments with n = 4–5 mice per group. *** p < 0.001, ns = not significant. (TIF) [file pone.0201249.s001.tif]

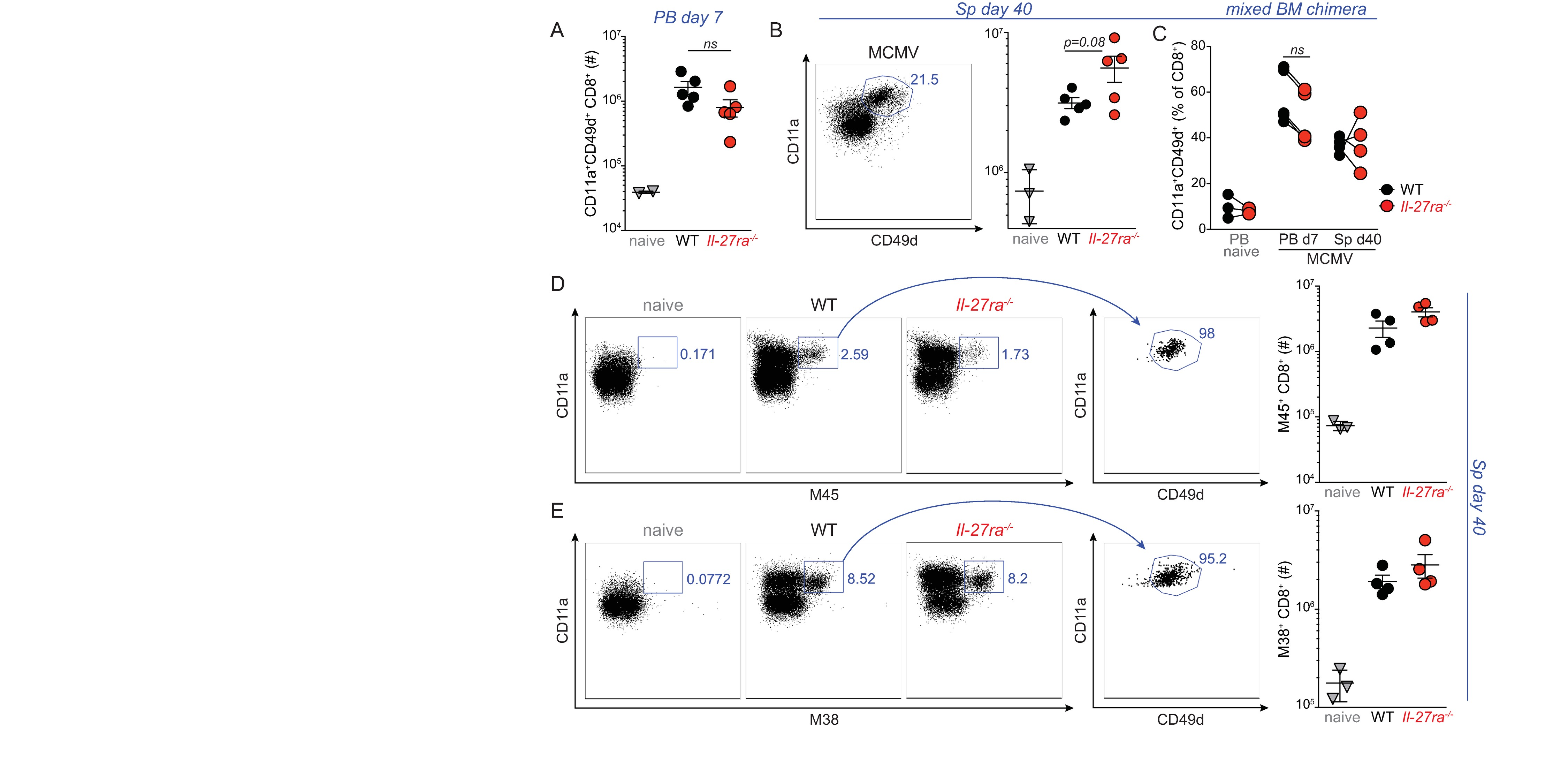

Supplement: S2 Fig — WT and Il27ra-/- mice were infected with 1*104 PFU MCMV and antigen experienced CD11a+CD49d+ CD8 T cells were enumerated in the blood at day 7 p.i. (A) and spleen at day 40 p.i. (B). (C) WT:Il27ra-/- chimeric mice were infected with 1*104 PFU MCMV and the proportion of CD11a+CD49d+ CD8 T cells within each compartment was analyzed in the blood (PB) of both uninfected and MCMV infected chimeric mice (day 7 p.i.) and in the spleen (Sp) of infected chimeras at day 40 p.i. (D-E). WT and Il27ra-/- mice were infected with 1*104 PFU MCMV and the number of M45985-993 (D) and M38316-323 (E) virus specific CD8 T cells was determined in the spleen at day 40 p.i. (A-B) one representative of 3 independent experiments with n = 4–5 mice per group. (C-E) one representative of 2 independent experiments with n = 4–5 mice per group. ns = not significant. (TIF) [file pone.0201249.s002.tif]

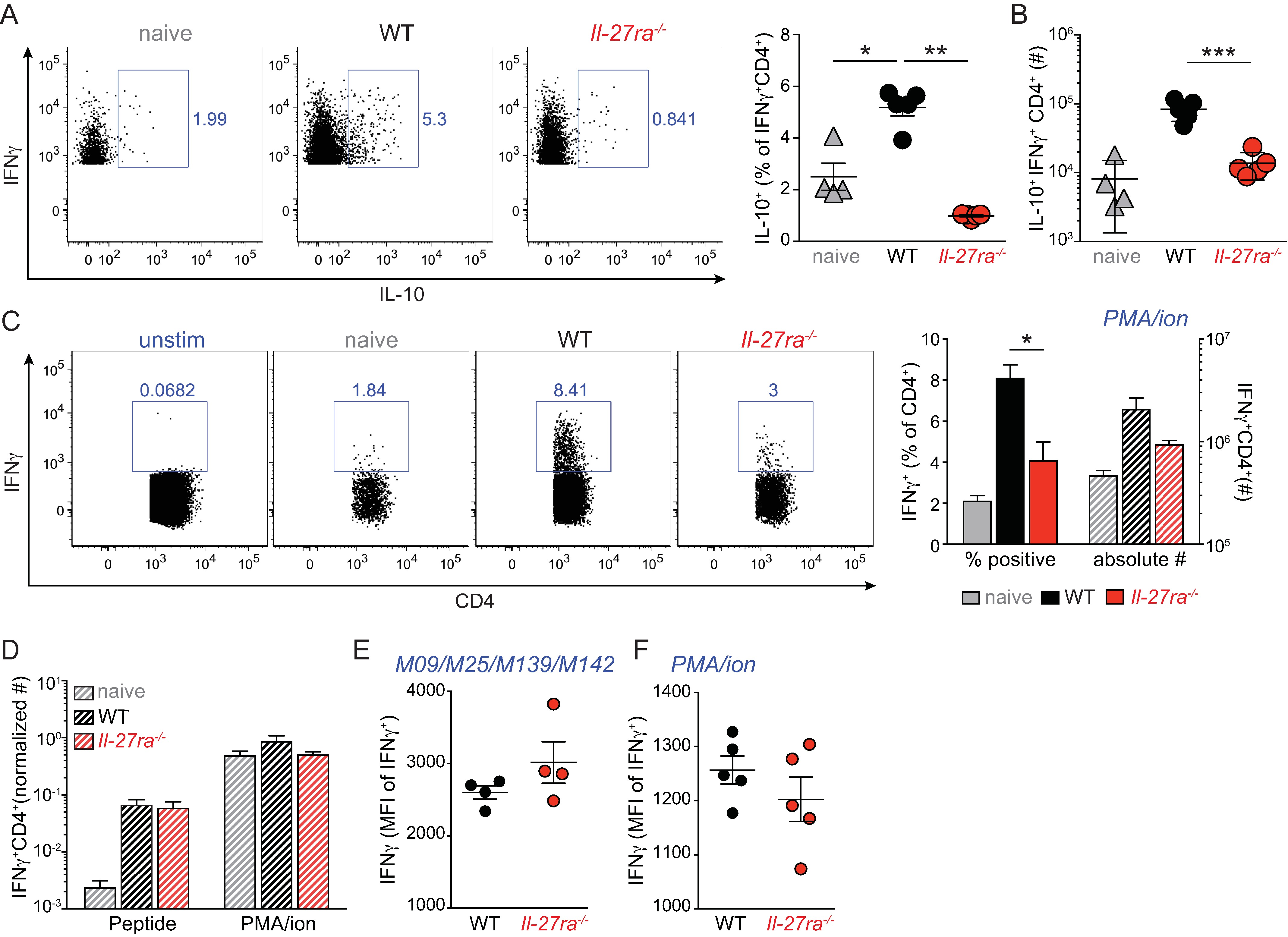

Supplement: S3 Fig — WT and Il27ra-/- mice were infected with 1*104 PFU MCMV and the proportion (A) and number (B) of IL-10+ cells within IFNγ producing CD4 T cells was analyzed upon PMA/ion stimulation in the spleen at day 21 p.i. (C) WT and Il27ra-/- mice were infected with 1*104 PFU MCMV and the proportion and number of IFNγ producing CD4 T cells were determined upon PMA/ion stimulation in the spleen at day 40 p.i. (D) The number of IFNγ producing CD4 T cells upon M09133-147, M25409-423, M139560-574 and M14224-38 peptide specific restimulation and polyclonal PMA/ion stimulation in the spleen at day 40 p.i. normalized for the total amount of polyclonal CD11a+CD49d+ CD4 T cells present. (E-F) IFNγ mean fluorescence intensity (MFI) in IFNγ+ CD4 T cells upon M09133-147, M25409-423, M139560-574 and M14224-38 peptide specific restimulation (E) and polyclonal PMA/ion stimulation (F) in the spleen at day 40 p.i. All data are representative of at least two independent experiments with n = 4–5 mice per group. * p < 0.05, ** p < 0.01, *** p < 0.001. (TIF) [file pone.0201249.s003.tif]

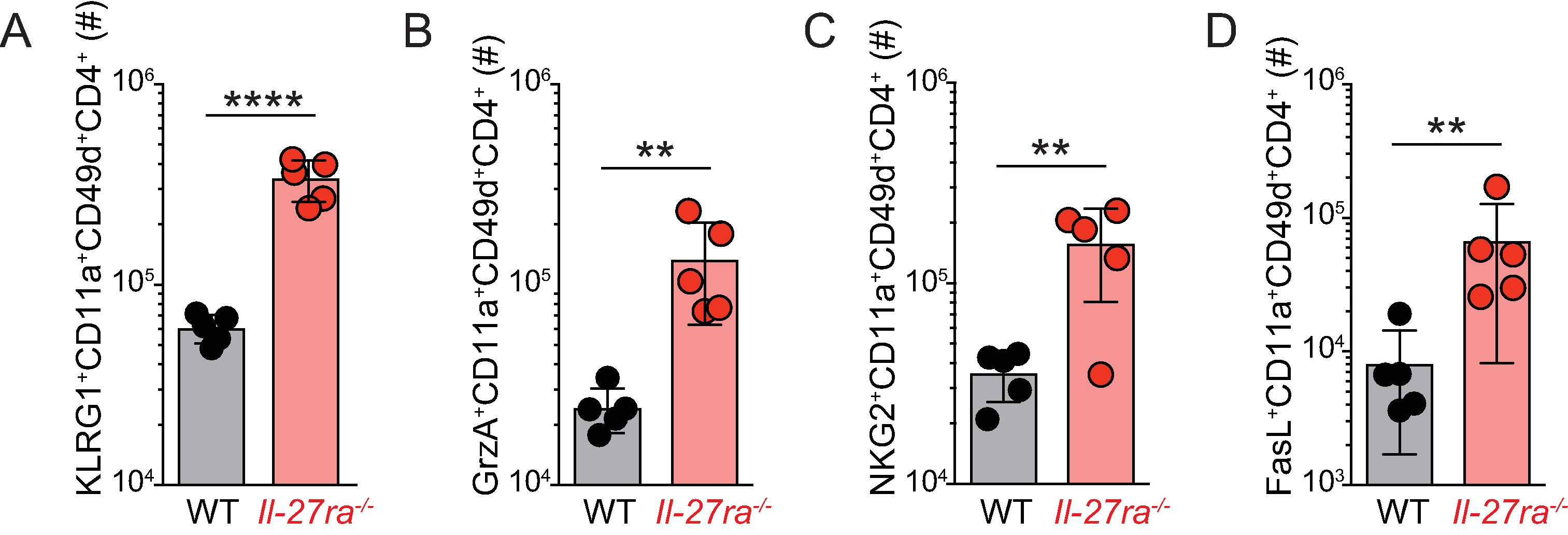

Supplement: S4 Fig — WT and Il27ra-/- mice were infected with 1*104 PFU MCMV and the number of polyclonal CD11a+CD49d+ CD4 T cells expressing KLRG1 (A), GrzA (B), NKG2A/C/E (C) and FasL (D) were determined in the spleen. All data are representative of at least two independent experiments with n = 5 mice per group. ** p < 0.01, ****p < 0.001. (TIF) [file pone.0201249.s004.tif]

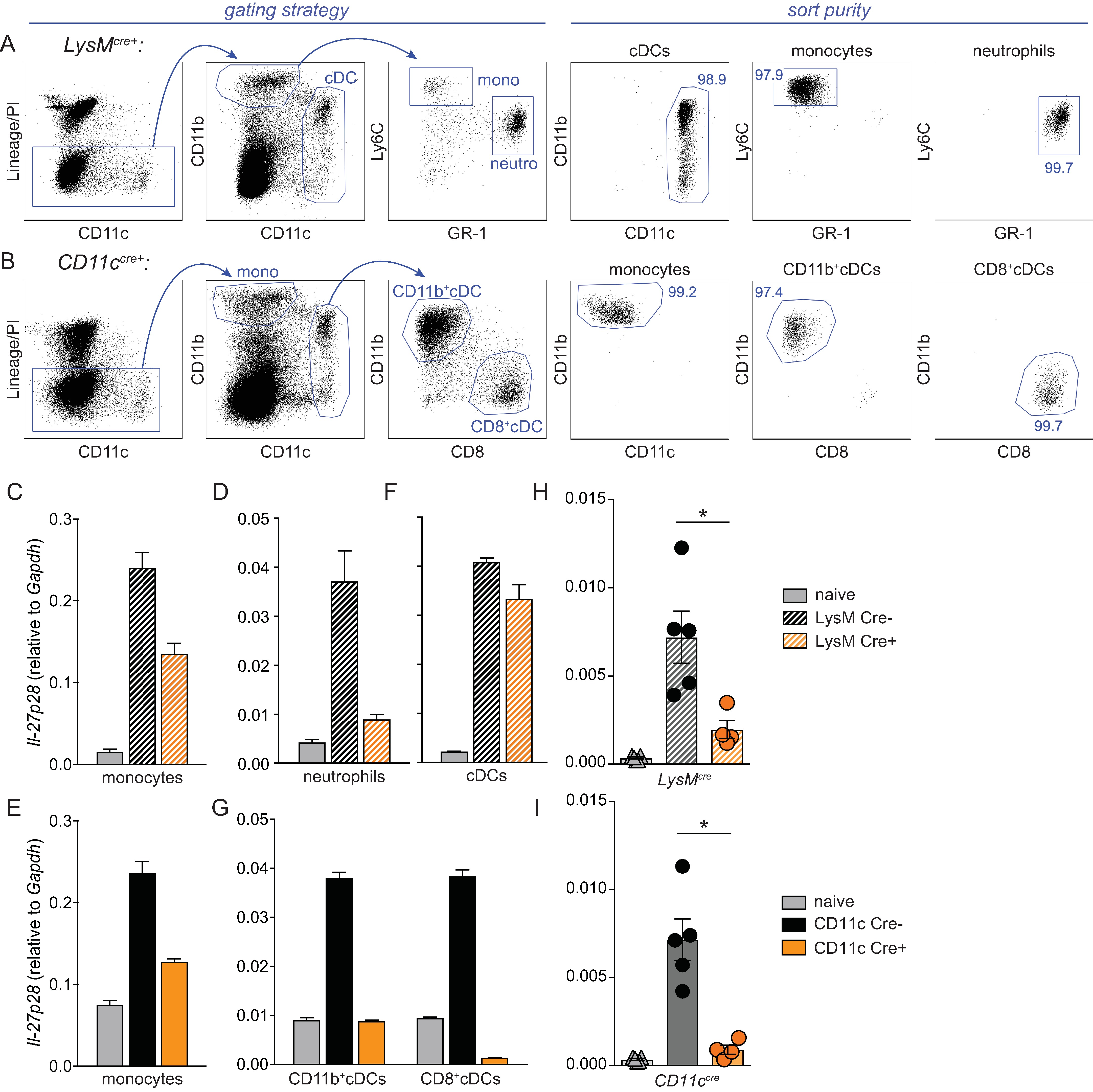

Supplement: S5 Fig — LysMcre+IL27p28fl/fl and CD11ccre+IL27p28fl/fl mice or cre- littermate controls were left untreated or infected with 1*104 PFU MCMV. 36 hours post infection innate cell populations were FACS purified from pooled spleen samples and the relative levels of Il27p28 over Gapdh were determined by qPCR. (A-B) gating strategy and post sort purity in LysMcre+IL27p28fl/fl mice (A) and CD11ccre+IL27p28fl/fl mice (B). Relative level of Il27p28 over Gapdh in sorted monocytes (PI-Thy1.2-CD19-NK1.1-Siglec-F-CD11c-CD11bhiLy6Chi) (C), neutrophils (PI-Thy1.2-CD19-NK1.1-Siglec-F-CD11c-CD11bhiGR-1+) (D) and cDCs (PI-Thy1.2-CD19-NK1.1-Siglec-F-CD11chi) (F) of infected LysMcre+IL27p28fl/fl mice compared to cre- and uninfected controls. Relative level of Il27p28 over Gapdh in sorted monocytes (PI-Thy1.2-CD19-NK1.1-Siglec-F-GR-1-CD11c-CD11bhi) (E) and CD11b+ (PI-Thy1.2-CD19-NK1.1-Siglec-F-GR-1-CD11chiCD11b+CD8-) and CD8+ (PI-Thy1.2-CD19-NK1.1-Siglec-F-GR-1-CD11chiCD11b-CD8+) cDCs (G) of infected CD11ccre+IL27p28fl/fl mice compared to cre- and uninfected controls. (H-I) Il27p28 transcript levels relative to Gapdh in spleen homogenates of LysMcre+IL27p28fl/fl mice (H) and CD11ccre+IL27p28fl/fl mice (I) at 36 hours p.i. compared to cre- and uninfected controls One representative of two independent experiments with pooled samples from n = 4–5 mice per group. (TIF) [file pone.0201249.s005.tif]

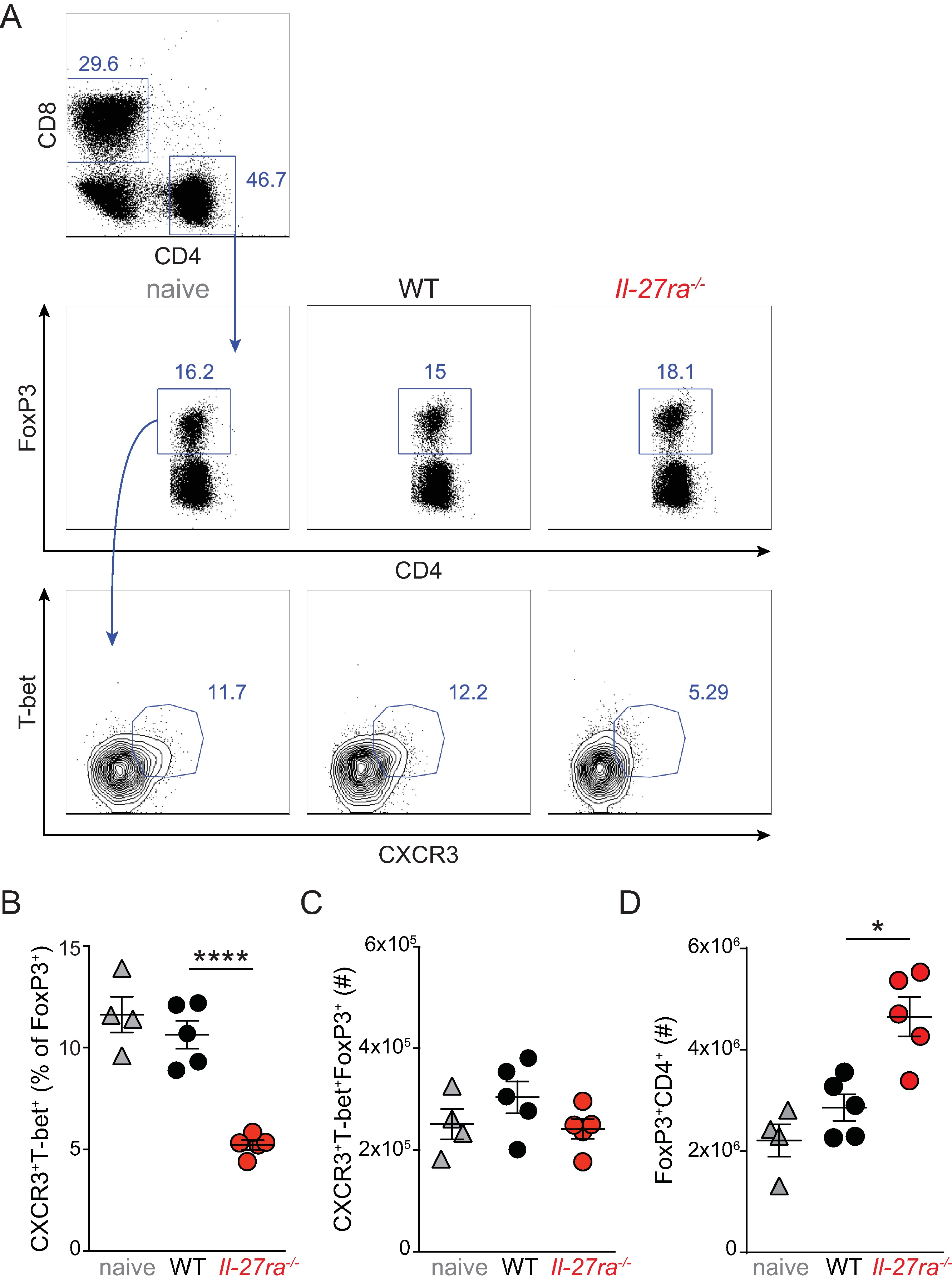

Supplement: S6 Fig — WT and Il27ra-/- mice were infected with 1*104 PFU MCMV. (A) Gating strategy applied to quantify CD4+CXCR3+T-bet+FoxP3+ Tregs. Proportion (B) and number (C) of CD4+CXCR3+T-bet+FoxP3+ Tregs and total number of CD4+FoxP3+ Tregs (D) analyzed in the spleen at d21 p.i. All data are representative of two independent experiments with n = 5 mice per group. * p < 0.05, ** p < 0.01. (TIF) [file pone.0201249.s006.tif]
